# Mutational and gene fusion analyses of primary large cell and large cell neuroendocrine lung cancer

**DOI:** 10.18632/oncotarget.4314

**Published:** 2015-06-17

**Authors:** Anna Karlsson, Hans Brunnström, Kajsa Ericson Lindquist, Karin Jirström, Mats Jönsson, Frida Rosengren, Christel Reuterswärd, Helena Cirenajwis, Åke Borg, Per Jönsson, Maria Planck, Göran Jönsson, Johan Staaf

**Affiliations:** ^1^ Division of Oncology and Pathology, Department of Clinical Sciences Lund, Lund University, Medicon Village, SE 22381 Lund, Sweden; ^2^ Division of Oncology and Pathology, Department of Clinical Sciences Lund, Lund University, SE 22185 Lund, Sweden; ^3^ Department of Pathology, Regional Laboratories Region Skåne, SE 22185 Lund, Sweden; ^4^ Department of Thoracic Surgery, Lund University, Skåne University Hospital, SE 22185 Lund, Sweden; ^5^ Department of Oncology, Skåne University Hospital, SE 22185 Lund, Sweden; ^6^ Create Health Strategic Center for Translational Cancer Research, Lund University, Medicon Village, SE 22381 Lund, Sweden

**Keywords:** large cell lung cancer, LCNEC, mutation, gene fusion, ALK

## Abstract

Large cell carcinoma with or without neuroendocrine features (LCNEC and LC, respectively) constitutes 3–9% of non-small cell lung cancer but is poorly characterized at the molecular level. Herein we analyzed 41 LC and 32 LCNEC (including 15 previously reported cases) tumors using massive parallel sequencing for mutations in 26 cancer-related genes and gene fusions in *ALK*, *RET*, and *ROS1*. LC patients were additionally subdivided into three immunohistochemistry groups based on positive expression of TTF-1/Napsin A (adenocarcinoma-like, *n* = 24; 59%), CK5/P40 (squamous-like, *n* = 5; 12%), or no marker expression (marker-negative, *n* = 12; 29%). Most common alterations were *TP53* (83%), *KRAS* (22%), *MET* (12%) mutations in LCs, and *TP53* (88%), *STK11* (16%), and *PTEN* (13%) mutations in LCNECs. In general, LCs showed more oncogene mutations compared to LCNECs. Immunomarker stratification of LC revealed oncogene mutations in 63% of adenocarcinoma-like cases, but only in 17% of marker-negative cases. Moreover, marker-negative LCs were associated with inferior overall survival compared with adenocarcinoma-like tumors (*p* = 0.007). No *ALK*, *RET* or *ROS1* fusions were detected in LCs or LCNECs. Together, our molecular analyses support that LC and LCNEC tumors follow different tumorigenic paths and that LC may be stratified into molecular subgroups with potential implications for diagnosis, prognostics, and therapy decisions.

## INTRODUCTION

Non-small cell lung cancer (NSCLC) accounts for the majority of diagnosed lung cancers and is dominated by the adenocarcinoma, squamous cell carcinoma (SqCC) and large cell carcinoma with or without neuroendocrine features (LCNEC and LC, respectively) histological subtypes. In NSCLC, LC and LCNEC together account for 3–9% of all cases depending on cohort demographics and classification scheme, with a generally poor prognosis compared to other NSCLC subgroups [1, 2]. In the 2004 WHO classification of lung cancer LC is defined as an undifferentiated NSCLC lacking architectural and cytologic features of small-cell carcinoma, glandular or squamous differentiation, whereas LCNEC is defined as an LC with neuroendocrine morphological features and at least one positive neuroendocrine immunohistochemical (IHC) marker [3]. LCNEC tumors share many similarities with small-cell lung cancer (SCLC) on the morphological, IHC and molecular level [4] (and references therein). Based on advances in immunomarkers for classification of adenocarcinoma and SqCC there is today significant controversy on whether LC actually represent a truly distinct biological entity, or merely a group of very poorly differentiated tumors of other NSCLC groups (adenocarcinoma and/or SqCC) [5, 6]. In fact, in the most recent 2015 WHO classification of lung cancer LCs that are mucin-positive or expresses pneumocyte markers should now be classified as adenocarcinoma, and the squamous marker-positive cases as nonkeratinizing squamous cell carcinoma [7].

In comparison to other NSCLC subgroups, LC and LCNEC tumors remain fairly uncharacterized at the molecular level by modern genomic techniques. Recent studies have investigated copy number alterations (CNAs) in LC and LCNEC [8, 9], highlighted the transcriptional similarity between LCNEC and SCLC [8], and identified a neuroendocrine DNA methylation subgroup in lung cancer [10]. In contrast, studies of the genome-wide mutational landscape in LC and LCNEC using massive parallel sequencing methods (NGS) are scarce. A recent analysis of 15 LCNEC tumors using whole-exome sequencing associated mainly mutations in *TP53*, *RB1*, and *EP300* with LCNEC (and SCLC) tumor histology, with additional mutations in LCNEC also found in adenocarcinomas and SqCCs [8]. Studies of smaller gene sets have identified abnormal *TP53* expression in both LC and LCNEC tumors and *KRAS* mutations predominantly in LCs [6, 11]. Mutations in *EGFR* and *ALK* gene fusions represent current molecular treatment predictive alterations for targeted therapy in lung cancer [12], but rarely appear in LC or LCNEC tumors with only a few reported cases in the literature [5, 6, 13–16]. Clearly, better characterization of the mutational landscape in LC and LCNEC is needed to take advantage of the growing number of targeted treatments and our emerging understanding of treatment resistance factors in lung cancer.

In this study, we aimed to investigate the mutational landscape of LC and LCNEC tumors using a panel of 26 well-established oncogenes and tumor suppressor genes in combination with *ALK*, *RET*, and *ROS1* gene fusion analysis and copy number analysis of targeted genes. To this end, we analyzed 41 LC and 17 LCNEC cases by massive parallel sequencing and combined our results with 15 whole-exome sequenced LCNEC cases [8] and previously reported copy number data [8, 10].

## MATERIALS AND METHODS

### Patient material

DNA and total RNA were extracted from 57 early stage lung cancer patients surgically treated at the Skåne University Hospital in Lund, Sweden (Table 1 and Supplementary Table S1). Patients in this retrospective study had not received any neoadjuvant treatment before surgery. One patient harbored a mixed cancer, with one LC and one LCNEC tumor component, treated as two individual tumors in the analysis. In total, 41 LC and 17 LCNEC samples were included from this patient cohort. For all cases, relevant pathological slides were re-evaluated and clinicopathological characteristics were updated to be in line with recent international criteria and guidelines [3, 17]. Thirteen cases have been described in previous studies [10, 18]. From Seidel et al. [8], we included whole-exome sequencing and copy number data on genes investigated in the experimental Lund cohort from 15 additional LCNEC cases (Table 1).

**Table 1:** Patient characteristics and clinicopathological data

	Lund	CLCGP [8]	All cases
**Histology**			
LC (basaloid)	41 (6) *, **	-	41 (6)
LCNEC	17*	15	32
**LC immunomarker profile**			
Adenocarcinoma-like	24 (59%)	-	24 (59%)
Squamous cell carcinoma-like	5 (12%)	-	5 (12%)
Marker null	12 (29%)	-	12 (29%)
**Tumor stage**			
I	29	7	36
II	19	6	25
III	8	2	10
IV	2	0	2
**Smoking history**			
Never-smokers	0	0	0
Smokers	34	11	45
Not available	24	4	28
**Gender**			
Female	31	6	37
Male	27	9	36
**Age (median & range)**	66 (47–82)	67 (47–80)	66 (47–82)
**Patients evaluable for**			
Mutations	58	15	73
*ALK*, *RET*, *ROS1* fusions	46	1***	47
Copy number alterations	46	10	56

* One patient had a mixed tumor with both an LC and LCNEC component.

** Basaloid (*n* = 6) and lymphoepithelioma-like (*n* = 1) cases are included in the LC sample numbers.

*** Evaluated for *ALK*/*RET*/*ROS1* fusions by FISH.

### Ethics statement

The study was approved by the Regional Ethical Review Board in Lund, Sweden (Registration no. 2004/762, 2008/702, 2007/445, and 2014/748).

### Immunohistochemistry

Cases with neuroendocrine morphological features were evaluated for IHC staining of the neuroendocrine markers chromogranin A, synaptophysin and CD56 (Supplementary Methods). At least 10% positive tumor cells were required for positive staining for these markers. In addition, LC cases were analyzed for IHC staining of CK5/P40 (squamous cell markers) and TTF-1/Napsin A (adenocarcinoma markers). Staining intensities for these markers were categorized as 0 (<1% positive tumor cells), 1 (1–10%), 2 (11–25%), 3 (26–50%), and 4 (>50% positive tumor cells). Similar to the recent 2015 WHO update on lung cancer [7], we classified a categorized intensity of ≥1 as positive for TTF-1 or Napsin A, and ≥ 2 as positive for CK5 or P40. A LC sample was classified as adenocarcinoma-like if a positive TTF-1 and/or Napsin A staining was observed. A LC case was classified as squamous-like if a positive CK5 and/or P40 staining was observed. IHC analyses are further described in Brunnström et al. [19] and Supplementary Methods.

### Mutational analysis

All Lund cases were analyzed by the NGS-based Illumina TruSight Tumor gene panel on a MiSeq instrument according to manufacturer's instructions (Illumina, San Diego, CA, US). Analyzed regions included a selected set of complete exons in 26 genes: *AKT1* (exon 2), *ALK* (exon 23), *APC* (exon 15), *BRAF* (exons 11, 15), *CDH1* (exons 8, 9, 12), *CTNNB1* (exon 2), *EGFR* (exons 18, 19, 20, 21), *ERBB2* (exon 20), *FBXW7* (exons 7, 8, 9, 10, 11), *FGFR2* (exon 6), *FOXL2* (exon 1), *GNAQ* (exons 4, 5, 6), *GNAS* (exons 6, 8), *KIT* (exons 9, 11, 13, 17, 18), *KRAS* (exons 1, 2, 3, 4), *MAP2K1* (exon 2), *MET* (exons 1, 4, 13, 15, 16, 17, 18, 20), *MSH6* (exons 5), *NRAS* (exons 1, 2, 3, 4), *PDGFRA* (exons 11, 13, 17), *PIK3CA* (exons 1, 2, 7, 9, 20), *PTEN* (exons 1, 2, 3, 4, 5, 6, 7, 9), *SMAD4* (exons 8, 11), *SRC* (exon 10), *STK11* (exons 1, 4, 6, 8), and *TP53* (exons 2, 3, 4, 5, 6, 7, 8, 9, 10, 11). DNA extraction was performed using the Qiagen GeneRead (Qiagen, Hilden, Germany) kit for formalin-fixed paraffin embedded tissue (FFPE), or by the Qiagen AllPrep kit for fresh frozen tissue (Supplementary Methods). Macrodissection of FFPE cases were performed when possible prior to DNA extraction. Alignment, quality filtering, variant calling, and variant annotation were performed using the standard MiSeq Reporter and VariantStudio analysis pipeline (Illumina). Only nonsynonymous variants with a quality score equal to 100 that passed the bi-directional sequencing quality filter in TruSight Tumor were considered. Read depths (X) for genes with detected variants varied between 3024–143140X (median 18624X, interquartile range 28450X).

### Gene fusion analysis

Analysis of *ALK*, *RET*, and *ROS1* gene fusions were performed using the RNA-based Archer FusionPlex ARR v2 kit (ArcherDX, Boulder, CO, US) and the MiSeq instrument (Illumina) (Supplementary Methods). The HCC78 (*ROS1*-*SLC34A2*), KARPAS-299 (*ALK*-*NPM1*), LC-2/ad (*CCDC6*-*RET*), and H2228 (*EML4*-*ALK*) cell lines were used as controls (Supplementary Methods). RNA from FFPE tissues (*n* = 11 samples) were extracted using the Qiagen Allprep FFPE extraction kit (Qiagen), while RNA from cell lines or fresh frozen tissue were extracted using the non-FFPE Qiagen Allprep extraction kit. Data analysis was performed using software tools provided with the Archer kit (ArcherDX). Confirmatory ALK immunohistochemistry was performed using the D5F3 antibody (Ventana Medical Systems), and confirmatory *ALK* FISH analysis using the Vysis ALK break apart FISH probe (Vysis) according to manufacturers' instructions.

### Copy number analysis

Calls of copy number gain, loss, amplification and focused copy number loss for genes included in the TruSight Tumor panel were made for 46 tumors in the Lund cohort based on data from ongoing or published studies on the same tumor cohort [10], and for ten cases from Seidel et al. [8] as described by [10, 20] and Supplementary Methods.

### NGS validation analyses

Ten mutations in *KRAS* detected by the TruSight Tumor panel were selected for validation by the Therascreen^®^ KRAS RGQ PCR Kit (Qiagen, Hilden, Germany) according to the manufacturer's protocol. In addition, seven unrelated tumor FFPE specimens, including two melanomas, two lung adenocarcinomas, and three colon cancers were also used to validate the NGS platform. These samples had verified mutations in *BRAF* (the two melanomas and one colon cancer: V600E), *KRAS* (two colon cancers: G13D and G12S), and *EGFR* (the two lung adenocarcinomas: L858R and E746_A750del). Mutations in these cases were obtained from routine clinical diagnostics based on pyrosequencing or Q-PCR performed at the Skåne University Hospital in Lund, Sweden.

## RESULTS

### Tumor and patient characteristics

A cohort of 41 LC and 17 LCNEC cases (Lund cohort) were pooled with 15 reported LCNEC cases [8], thus rendering a total of 73 cases (Table 1). All patients with available chart data were (current or former) smokers. There were no statistical differences in the distribution of tumor stage, gender, or age of diagnosis between LC and LCNEC cases (*p* > 0.05, Fisher's exact test or Wilcoxon's test). Among the LC cases, six tumors were histopathologically subclassified as basaloid, and one as lymphoepithelioma-like. Protein expression of adenocarcinoma markers TTF-1 and Napsin A and squamous markers CK5 and P40 were investigated in the Lund cohort and the public LCNEC cohort (TTF-1 only). 77% of all analyzed LCNEC cases showed positive TTF-1 expression. 59% of LC cases in the Lund cohort were IHC positive for TTF-1, while 44% were IHC positive for Napsin A. 75% of the TTF-1 positive LC cases also showed positive Napsin A expression, while no case was Napsin A positive but TTF-1 negative. For the squamous markers CK5 and P40, 5% and 10% of LC cases showed positive staining, respectively.

### The mutational spectrum of LC and LCNEC

**Figure 1:**
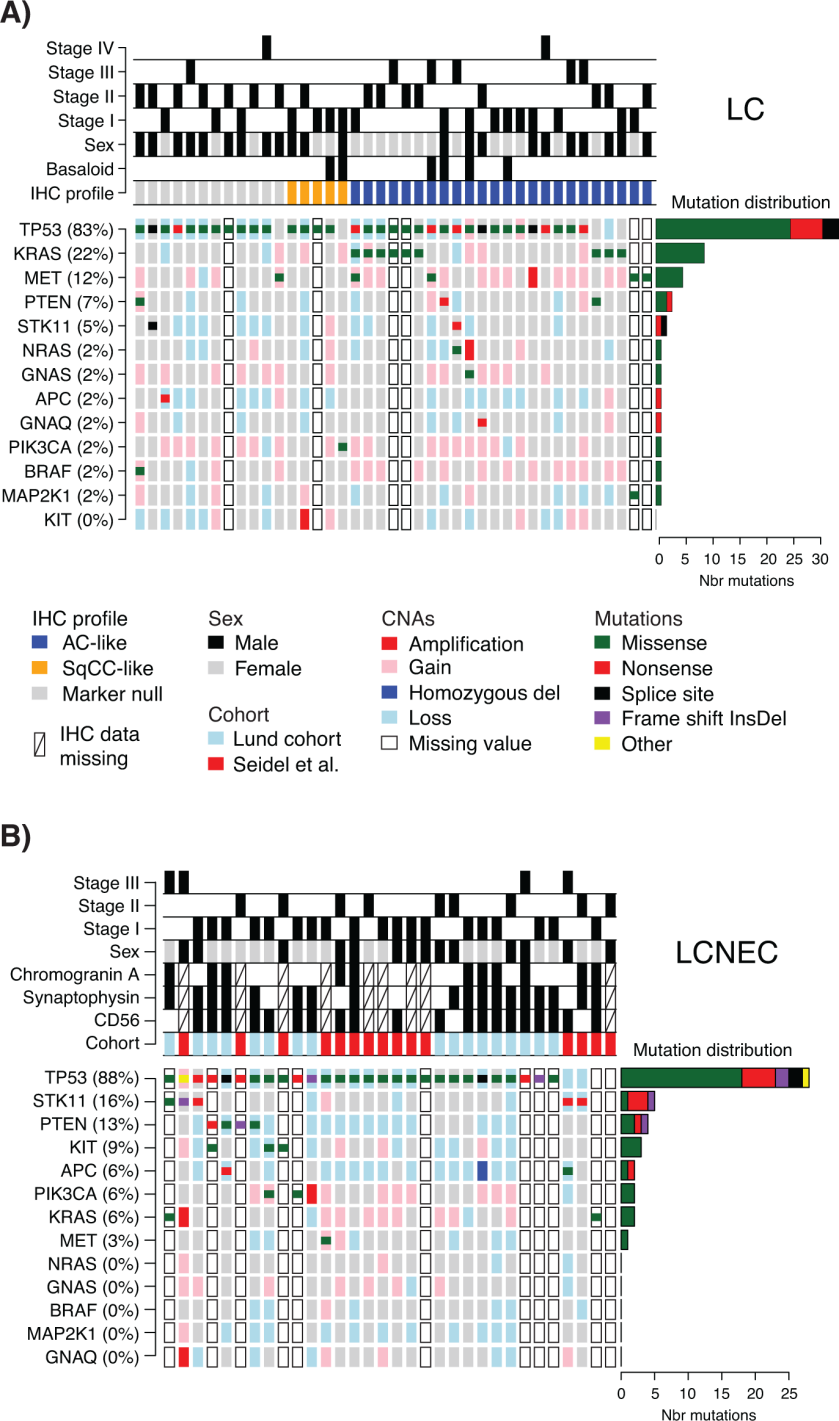
Detected mutations and copy number alterations in LC and LCNEC **A.** Detected gene variants and copy number alterations (CNAs) (rows) in 41 LC cases (columns), ordered by immunomarker profile of adenocarcinoma-like (AC-like), squamous cell carcinoma like (SqCC-like), or marker null phenotype (TTF-1/Napsin A and CK5/P40 negative). Copy number status is shown as larger background rectangles and mutations as squares for each sample and gene. Right side bar plot summarizes the distribution of the different mutation types for each gene. **B.** Detected variants and copy number alterations in 32 LCNEC cases displayed as in A. Samples are ordered according to gene variant frequency.

The 73 LC and LCNEC cases were analyzed for mutations in 26 cancer-related genes through NGS-based analysis of fresh frozen or FFPE tumor tissues. In total, 117 nonsynonymous variants, with alternate variant frequencies (the fraction of all reads with the detected variant) between 3–91% (Lund cohort only), were identified in 13 genes, for which gene copy number status were also extracted (Figure 1 and Supplementary Figure S1 and Supplementary Tables S2–S3 listing explicit variant data). Median number of variants per sample was one and maximum was three. 72 out of 73 cases, including all Lund cases, showed variants in at least one gene.

*TP53* mutations were the most dominant alteration in both LC and LCNEC tumors (83% and 88% of cases, respectively). *TP53* mutations typically manifested as missense mutations in active protein domains and nonsense mutations in between active domains (Figure 1 and Supplementary Figure S1). Remaining alterations were found in considerably lower numbers in both subgroups. In LC, *KRAS* and *MET* were the second and third most frequently mutated genes (22% and 12%, respectively), while corresponding genes in LCNEC were *STK11* and *PTEN* (16% and 13%, respectively) (Figure 1, Supplementary Table S3). These alterations highlight a more general difference between the two subgroups, regarding alterations in oncogenes versus tumor suppressors. Here, LC cases typically showed more alterations in oncogenes compared to LCNECs. Specifically, 20 oncogene alterations in *BRAF*, *GNAQ*, *GNAS*, *KRAS*, *KIT*, *MET*, *NRAS*, *MAP2K1/MEK1*, and *PIK3CA* were found in 44% of the LC cases as compared to eight alterations affecting 22% of LCNEC cases (Supplementary Table S3). Notably, for the two *KRAS* alterations observed in LCNEC cases, one was not in the active RAS protein domain (a *KRAS* M1I mutation). The second, a G12C mutation, was found in both the LCNEC and the LC component of the included multicomponent tumor, with different alternate allele frequencies (40.3% in the LCNEC and 7.9% and in the LC component of the tumor). Together, this suggests that activating *KRAS* mutations are in fact rare in LCNEC. Consistent with a general idea of a limited number of oncogene hits required to activate a tumorigenic pathway, we observed only one case in each histological subgroup with >1 mutation in any of the eight oncogenes.

Two additional differences regarding oncogene mutations may be noted. Firstly, *KIT* mutations were exclusively found in LCNEC cases (*n* = 3, 9%). Secondly, all *TP53* wild type LC cases (17% of all LC cases) harbored oncogene mutations in *KRAS*, *MET* or *PIK3CA* (Figure 1A). This observation suggests that these *TP53* wild type tumors may be more dependent on oncogene activation alone for sustained tumor development. However, in this relatively small retrospective cohort we did not find support for differences related to tumor stage or gender (*p* = 0.69 and *p* = 0.42, respectively, Fisher's exact test), or patient outcome (overall survival, log-rank *p* > 0.05) between these tumors and *TP53*-mutated LCs.

Finally, high-level copy number gain (amplifications), or low copy deletions (putative homozygous deletions) were very scarce in analyzed cases (only single cases with *NRAS*, *KRAS*, *GNAQ*, *MET*, *KIT*, or *PIK3CA* amplifications, Figure 1), and no distinct cases of monoallelic amplification of mutated oncogene alleles were observed. For several tumor suppressors (*TP53*, *STK11*, *PTEN*, and *APC*), in especially LCNEC cases, we observed apparent support of Knudson's multiple-hit hypothesis [21], with DNA mutation and associated copy number loss (Figure 1).

### DNA mutations in histopathological and immunohistological subgroups of LC and LCNEC

Basaloid tumors represent a rare histopathological subgroup of LC characterized by specific cytological and tissue architectural characteristics [3]. In our LC cohort, six cases were subclassified as basaloid cancer. When viewed as two subgroups, i.e., basaloid versus non-basaloid LC, there were no differences in mutation frequencies between the groups for two of the most commonly mutated genes, *TP53* and *MET*. In contrast, no *KRAS* mutations were observed in basaloid cases, consistent with Rossi et al. [6].

**Figure 2:**
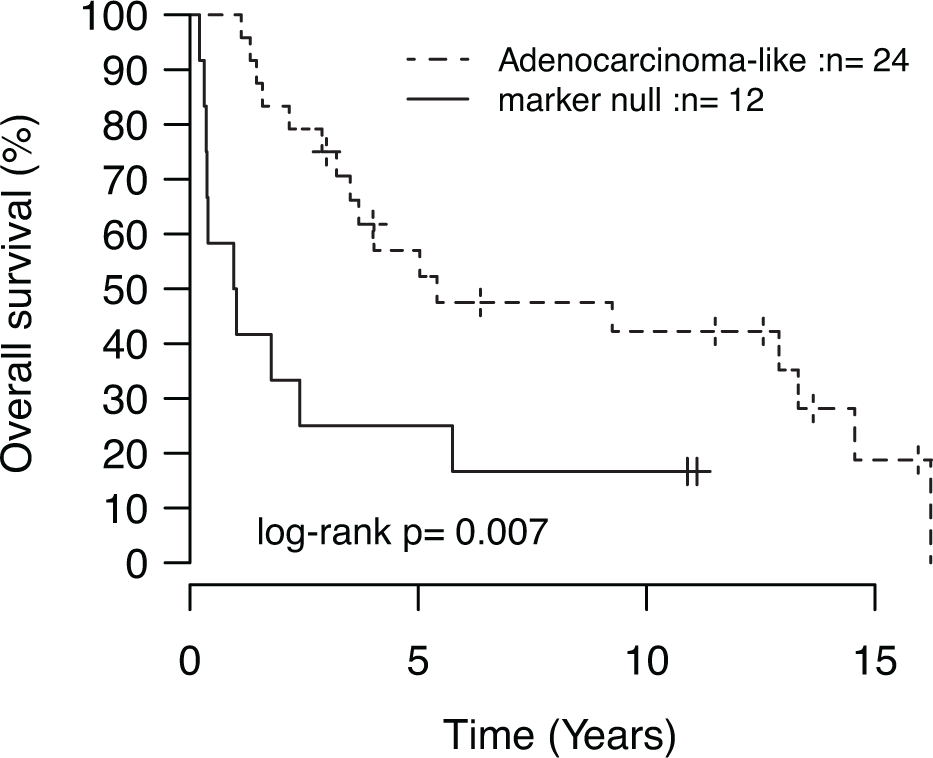
Kaplan-Meier analysis of the association with overall survival for immunomarker-defined subgroups of LC *P*-value calculated using the log-rank test.

Recently, patient outcome and specific oncogene mutations in LC tumors have been associated with tumor subgroups defined by positive expression of adenocarcinoma (TTF-1/Napsin A) or squamous cell carcinoma (CK5/P40) immunohistochemistry markers [5]. In our LC cohort, 24 tumors (59%) were positive by immunohistochemistry for TTF-1/Napsin A (referred to as adenocarcinoma-like), 5 tumors (12%) were CK5/P40 positive (squamous-like), whereas 12 tumors (29%) did not express any of these IHC markers (marker null cases). Stratification of identified mutations by IHC subgroup revealed a striking enrichment of oncogene mutations in adenocarcinoma-like LC tumors (85% of all oncogene mutations, affecting 63% of these cases), including all nine *KRAS* mutations and the single *NRAS* (G12D) and *MAP2K1*/*MEK1* (K57N) mutations (Figure 1A, Supplementary Tables S2 and S3). These *KRAS* mutations were all typical driver mutations located in codon 12 (one G12S, two G12C, three G12V mutations), 13 (one G13C and one G13D mutation), and 61 (one Q61K mutation), suggesting that these represent likely driver events in the affected tumors. In contrast, 92% of marker null cases carried a *TP53* mutation, but only 17% of cases had an oncogene mutation (one *BRAF* Q456K and one *MET* T1010I mutation). Moreover, marker null cases showed a poorer overall survival compared to adenocarcinoma-like cases (*p* = 0.007, log-rank test, Figure 2). The poorer outcome of marker null LC cases compared to adenocarcinoma-like cases was significant also in multivariate analysis including immunomarker stratification, tumor stage, and gender as covariates and overall survival as clinical endpoint (Hazard ratio = 4.4, 95% Confidence interval = 1.5–12.5, *p* = 0.006 for marker null stratification).

For LCNEC cases, we observed no association between DNA mutations and IHC expression of the chromogranin A, synaptophysin or CD56 neuroendocrine markers.

### Validation analyses of NGS DNA mutation results

To validate the NGS platform we first analyzed seven independent tumor FFPE samples from lung, colon and melanoma with known mutations in *BRAF* (V600E), *KRAS* (G12S, G13D), and *EGFR* (L858R and E746_A750del). The variant allele frequency for these alterations by pyrosequencing ranged between 27.7–41.8%. All mutations could successfully be identified by the NGS platform.

Secondly, we selected the ten LC and LCNEC cases from the Lund cohort harboring *KRAS* mutations (variant allele frequencies between 3.1–41%), and validated them using quantitative PCR (Qiagen Therascreen). Eight *KRAS* mutations were correctly identified, while the remaining two, Q61K and G13C, were not covered by the Therascreen assay.

### ALK, RET, and ROS1 gene fusion analysis in LC and LCNEC

The ability of the Archer FusionPlex assay to identify gene fusions in *ALK*, *RET* and *ROS1* was successfully validated in four cell lines with known fusion gene rearrangements, HCC78 (*ROS1*-*SLC34A2*), KARPAS-299 (*ALK*-*NPM1*), LC-2/ad (*CCDC6*-*RET*), and H2228 (*EML4*-*ALK*).

Fusion gene analysis was performed on all 58 Lund cases using RNA from fresh frozen (*n* = 47) or FFPE (*n* = 11) tumor tissues. However, only 46 fresh frozen tumors passed the initial Archer data quality analysis steps after sequencing (35 LC, 11 LCNEC). The failure of the FFPE cases is likely due to extensive RNA degradation in the tissue blocks caused by the fixation process and subsequent storage. In the 46 analyzable cases, we identified no *RET* or *ROS1* fusions. Only one analyzed tumor, a LCNEC case, showed a candidate *ALK* gene fusion event (*DNBL*-*ALK*) based on NGS data, however just with the minimum number of reads required for reporting (Supplementary Methods). However, confirmatory ALK IHC and FISH analysis could not confirm protein overexpression or an actual gene fusion event in this case.

## DISCUSSION

In the current study, we have explored the mutational spectrum of 26 well-established cancer-related genes and *ALK*, *RET*, and *ROS1* gene fusions by massive parallel sequencing in a large panel of thoroughly histopathologically classified primary LC and LCNEC lung cancers. In comparison to existing methods, NGS-based methods for DNA variant detection generally offer higher sensitivity in detecting low-frequency variants. Together with the specific feature of bi-directional sequencing in the Illumina TruSight Tumor assay this allows for sensitive variant detection also in FFPE samples. Besides the presented molecular characterization of LC and LCNEC tumors, the current study also supports the feasibility of using NGS-based methods for analysis of treatment predictive DNA alterations in routine clinical lung FFPE tumor tissues.

In LC as a whole, our findings of frequent *KRAS* mutations and less frequent alterations in *BRAF*, *MAP2K1*, and *PIK3CA* are in agreement with previous studies [5, 6]. Similarly, in LCNEC the high mutation rate of *TP53* and the scarcity of *KRAS* mutations have also been reported before (see, e.g., Rossi et al. [4]). The similar frequency of *TP53* mutations between the LC and LCNEC group mimics findings of similar p53 protein expression by Iyoda et al. [11]. Due to a paucity of studies, the roles of *PTEN*, *STK11* and *MET* mutations in LC and LCNEC are largely unknown. In our study, alterations in the tumor suppressors *PTEN* and *STK11* were mainly observed together with *TP53* mutations in both LC and LCNEC, while *MET* mutations were more often found in *TP53* wild type LC cases. Although LCNEC tumors have been shown to strongly express receptor tyrosine kinases such as *KIT*, *PDGFRA*, *PDGFRB* and *MET*, compared to other NSCLC groups, there is less support of mutations being the underlying cause for the elevated expression [4, 18]. Supporting these results, we identified no *PDGFRA* mutations in any of the tumors, only one *MET* mutation in the LCNEC group, whereas three *KIT* mutations were found exclusively in LCNEC cases. However, the impact of some of these mutations is difficult to assess without functional characterization, as all do not occur in active protein domains (see Supplementary Figure S1).

In this study, alterations in oncogenes, with exception of *KIT* alterations, are generally more frequent in LCs when considered a single entity compared to LCNEC. However, it is becoming apparent that the mutational spectrums in LC and LCNEC are different based on recent whole-exome sequencing studies. Specifically, LCNEC has been suggested to be more similar to SCLC [8], in line with the similarity of LCNEC and SCLC on the morphological, immunohistochemical, transcriptional, copy number, and epigenetic levels [4, 8–10]. Consistently, SCLC have recently been reported to harbor high frequencies of *TP53* mutations and similar frequencies of *KIT*, *PIK3CA* and *KRAS* alterations as for the LCNEC cases in the current study [22]. Together, our observations further support that LC and LCNEC follow different evolutionary paths.

Stratification of LC cases based on immunomarkers for adenocarcinoma (TTF-1/Napsin A) and squamous cell carcinoma (CK5/P40) revealed that 71% of the cases could be classified as variants of adenocarcinoma or SqCC. This observation is in line with previous reports (59–90%) [5, 23–27], although the observation of 29% of LC as marker null is on the higher end compared to the literature. One reason for this could be that we in this retrospective cohort used the TTF-1 8G7G3/1 clone that compared to the SPT24 clone is slightly less sensitive, whereas the SPT24 clone yields more cases positive for both CK5/P40 and TTF-1. Likewise, we used CK5 and P40 as markers of squamous cell carcinoma, while P63 may be more sensitive (but also less specific). Irrespectively, our data demonstrates the value of using multiple immunomarkers for undifferentiated lung cancers. While no apparent differences in oncogene amplification frequency could be observed between immunomarker-defined subgroups, the subgroups showed a distinctively different spectrum of especially oncogene mutations. Adenocarcinoma-like LCs (59% of all analyzed LC cases) harbored the overwhelming majority of detected oncogene mutations (85%), affecting 63% of these tumors. By comparison, only 17% of marker null LC cases showed oncogene mutations. Thus, adenocarcinoma-like LC appears to represent a more oncogene driven subgroup compared to CK5/P40 positive tumors and marker null LCs. These findings are in excellent agreement with Rekhtman et al. [5], including the observation of the single *PIK3CA* mutation in a CK5/P40 positive case, and a poorer overall survival for marker null patients compared to TTF-1/Napsin A positive LC patients. Despite the retrospective nature of the patient material, our results in combination with other recent molecular studies clearly challenge LC as an independent tumor entity on the molecular level, supporting that the current LC definition rather includes a heterogeneous collection of poorly differentiated tumors from other NSCLC subgroups [5, 6, 8]. In fact, supported by both clinicopathological and molecular studies the recent 2015 WHO classification of lung cancer now stress that the term LC should now only be used for undifferentiated tumors not expressing pneumocyte or squamous markers [7]. Importantly, in the 2004 WHO classification the LC definition provides little molecular information for a predictive molecular testing strategy to guide individualized treatment for this patient cohort [5]. A refined stratification of LC based on molecular characteristics may therefore have considerable impact on diagnosis, predictive molecular testing and in the end, therapy selection [5, 6, 8].

In contrast to the immunomarker-defined LC subgroups, less is known whether LCNEC tumors may be divided into similar subgroups. In the current study we found no associations of the neuroendocrine markers used to identify LCNEC tumors with specific mutations. This lack of association may be because these markers do not represent putative subgroups at all, and/or, as indicated by our analyses, that the mutational landscape in LCNEC is different in respect to, especially, oncogene drivers compared to non-neuroendocrine NSCLC. Clearly, further genomic studies of both marker null LC (undifferentiated LC) and LCNEC tumors including large scale sequencing approaches, gene expression profiling, and DNA methylation profiling are needed to further characterize these tumor groups.

*EGFR* mutations and *ALK* gene fusions are the key molecular treatment predictive alterations for targeted therapy in lung cancer today [12]. However, both alterations are scarce in LC and LCNEC tumors [5, 6, 14–16], consistent with our findings of no *EGFR* mutations or validated *ALK* gene fusions in either LC or LCNEC tumors. Specifically, the absence of *ALK* rearrangements in our LC cohort compared to the few *ALK* rearranged cases reported by Rekhtman et al. [5] is consistent with that our cohort comprises only of known smokers, while *ALK* rearrangements in the former study were found in never or light smokers. In recent studies, lung cancer patients with tumors harboring *ROS1* or *RET* gene fusions have shown notable responses to ALK or other multi-target kinase inhibitors [28, 29]. Similar to *ALK* fusions, the frequency of these alterations in LC and LCNEC is largely unknown, but may be expected to be very low. Consistently, we found no *RET* or *ROS1* gene fusions in LC or LCNEC tumors based on targeted RNA sequencing.

In summary, the current study adds further insights into the mutational landscape of LC and LCNEC, supporting that these tumor subgroups follow different tumorigenic paths. Moreover, our study supports that LC may be refined by molecular and immunomarkers into clinically relevant subgroups that may have implications for diagnosis, and therapy decisions. Despite the identification of adenocarcinoma-like LC as a subset of tumors with a potentially high frequency of forthcoming therapeutically relevant driver mutations, a continued search for additional molecular targets for therapeutic inhibition in non-adenocarcinoma NSCLC is warranted.

## SUPPLEMENTARY METHODS FIGURE AND TABLES








