# Supplementary material for: Mutational and gene fusion analyses of primary large cell and large cell neuroendocrine lung cancer
Source: Oncotarget. 2015 Jun 17;6(26):22028–37. doi: 10.18632/oncotarget.4314 (PMC4673143; doi:10.18632/oncotarget.4314)
Supplement: Supplementary file 1 [file oncotarget-06-22028-s001.pdf]

SUPPLEMENTARY METHODS

Lund patient cohort

For patients operated at the Skåne University Hospital in Lund, Sweden smoking history was obtained from patients' medical records and categorized into two groups; smokers (current and former) or never smoker. Follow-up data was obtained from the Swedish Cause of Death Register. For all cases, all relevant slides were reviewed for re-evaluation and updating of the histological diagnoses and stages to be in adherence with recent international criteria and guidelines [1–4].

External tumor cohort

Whole-exome sequencing and copy number data from 15 LCNEC cases were included from Seidel et al. [5]. Only genes where mutations were found in the experimental Lund cohort were included from these 15 LCNEC cases in the analyses.

Cell culturing and DNA/RNA extraction

The human non-small cell lung cancer (NSCLC) cell lines NCI-H228 (CRL-5935) and HCC-78 (ACC563) were purchased from the American Type Culture Collection (ATCC, USA) or the German Collection of Microorganisms and Cell Cultures (DSMZ, Germany), respectively. The human lung adenocarcinoma cell line LC-2/ad (06072604) and the Non-Hodgkin's Ki-positive Large Cell Lymphoma KARPAS 299 (94072247) were obtained from the European Collection of Cell Cultures (ECACC, UK) and further distributed by Sigma-Aldrich (Sweden). All cell lines, except for LC-2/ad were cultured in RPMI1640 medium supplemented with 10% fetal bovine serum (FBS). Note, the medium for the HCC-78 cell line was supplemented with heat-inactivated FBS instead. The LC-2/ad cell line was cultured in DMEM/HAMSF12. In addition, all cell lines grew in the presence of glutamine (2 mM) and penicillin (100 U/ml) and streptomycin (100 μg/ml). NCI-H228, HCC-78 and LC-2/ad cell lines were cultured in monolayers, whereas the KARPAS 299 cell line was grown in suspension. Cells were seeded into Petri dishes and harvested before reaching complete confluence by lysing the cells using RLT lysis buffer (Qiagen) and scraping. Cells were snap-frozen and stored at –80°C until extraction. DNA (and RNA) was extracted using the AllPrep DNA/RNA mini Kit (Qiagen, cat no 80204).

Immunohistochemistry

All stainings were performed on 4 μm thick sections from paraffin-embedded tissue previously fixed in 4% neutral aqueous formaldehyde for typically 48 h. The sections were from whole blocks (n = 42; cases from before 2005) or tissue microarrays (n = 16; cases from 2005–2011) with tumor and were automatically pre-treated and stained in a Ventana Bench-Mark Ultra (Ventana Medical Systems, Tucson, AZ). Control tissue for the immunohistochemical stains was used on each slide. Technical details for the antibodies and protocols are found in the table below. The staining results were evaluated by a pathologist (H.B.), and at least 10% positive tumor cells were required for positive staining (CD56, Chromogranin, Synaptophysin). LC and LCNEC cases were analyzed for IHC staining of CK5/P40 (squamous cell markers) and TTF-1/Napsin A (adenocarcinoma markers) (TTF-1 only for LCNEC). Staining intensity for these markers was categorized as 0 (<1% positive tumor cells), 1 (1–10%), 2 (11–25%), 3 (26–50%), and 4 (>50%). Special care was taken not to interpret trapped alveolar epithelium, macrophages etc. as tumor cells.

Antibody	Clone	Company	Dilution	Pre-treatment	Control tissue
CD56	123C3	Dako	1:50	CC1	Appendix (enteric nerves), tonsil (NK-cells)
Chromogranin A	LK2H10	Ventana	RTU	CC1	Pancreas, ileum (neuroendocrine cells)*
CK5	XM26	Leica	1:25	CC1	Esophagus, basal cells of bronchiole
Napsin A	IP64	Leica	1:20	CC2	Lung incl. terminal bronchiole, kidney
Synaptophysin	SP11	Ventana	RTU	CC1	Pancreas, ileum (neuroendocrine cells)*
P40	BC28	Biocare Medical	1:50	CC1	Esophagus, basal cells of bronchiole
TTF-1	8G7G3/1	Ventana	RTU	CC1 + Amp.	Lung incl. terminal bronchiole

*For chromogranin A and synaptophysin, enteric nerves (appendix) was also used as control tissue, but not on all slides used in the present study

Abbreviations: Amp, Amplification; CC1, Ventana Cell Conditioning 1 (EDTA, pH 8); CC2, Ventana Cell Conditioning 2 (citrate, pH 6); CK, cytokeratin; RTU, Ready to use; TTF-1, thyroid transcription factor 1

Extraction, quantification and qualification of nucleic acids

Nucleic acids from fresh frozen tumor tissue and formalin-fixed paraffin embedded tissue (FFPE) were extracted from 57 LC and LCNEC cases. One patient harbored a mixed cancer of both LC and LCNEC histology and was treated as two individual tumors. The two components were extracted and analyzed separately. A full section of the two components was also retrieved for comparison. FFPE blocks were macrodissected using H&E stains as guidance for selecting areas of the tumor with the highest tumor cell content. Excess paraffin and non-tumor cells were hereby excluded from the extraction process. Sectioning was performed using 5 µm thickness and 2–5 sections were used for separate DNA and RNA extractions. In an attempt to extract DNA and RNA from a similarly representative piece of the FFPE block, every other section was used for the two separate extractions. Sections that were not extracted immediately were stored at –80°C. DNA was extracted from FFPE tissue using the Qiagen GeneRead DNA FFPE Kit (cat no 180134) with modifications. Modifications included de-paraffinization using xylen instead of the de-paraffinization solution included in the kit and extension of the proteinase K digestion. DNA and RNA from fresh frozen tissue were extracted using a modified protocol of the AllPrep DNA/RNA Mini Kit (Qiagen). Quantification of RNA and DNA was performed using the Qubit® 2.0 System (Invitrogen). Quality of FFPE DNA was assessed according to manufacturer's instructions (TruSight Tumor Sample preparation, part# 15038313 Rev A., Illumina). During this process, a qPCR is performed to determine the amplifiability of the template, i.e. the FFPE DNA, compared to a supplied control template from which a ΔCt is calculated. This ΔCt value is a predictor of how well the extracted FFPE DNA will perform in the downstream library preparation and is used to the determine the DNA input amount for the library preparation. ΔCt values in the current study varied from 1.72 to 8.52 for the extracted FFPE DNA.

TruSight Tumor library preparation and sequencing

The TruSight Tumor panel enables detection of variant allele frequency below 5% in 174 amplicons across 26 genes. The panel is optimized for FFPE tissue and uses bi-directional library preparation and sequencing to overcome the issue of nucleic acid modification introduced by formalin-fixation. Only nucleic acid changes in both libraries for the same patient are considered as true variants. TruSight Tumor Sample Preparation (Cat no FC-130–2001, Illumina) was performed according to manufacturer's instructions on DNA extractions from fresh frozen tissue and FFPE tissue. Quantity of the prepared library was assessed using the Qubit® 2.0 System and a size estimate was retrieved from traces on High Sensitivity DNA Chips using the Agilent 2100 Bioanalyzer (Agilent Technologies). All libraries were normalized to 4 nM, denatured and sequenced on the MiSeq system (Illumina) using paired-end V2 chemistry.

ALK, RET and ROS1 fusion detection

100 ng RNA from fresh frozen tumors and 250 ng RNA from FFPE tissue was used as input to the Archer™ FusionPlex *ALK*, *RET*, *ROS1* Panel V2. Library preparation to detect known recurrent gene fusions as well as previously unidentified fusions was performed according to manufacturer's instructions. Libraries were quantified using the Qubit system, normalized to 4 nM, denatured and sequenced on the MiSeq system (Illumina) using paired-end V2 chemistry. Resulting FASTQ files from the sequencing was uploaded to the Archer Analysis Pipeline (http://archerdx.com/software/analysis)

Fusion gene validation

One candidate fusion gene event (an *DNBL-ALK*) categorized as a strong candidate by the Archer analysis pipeline was detected with the RNA panel. However, the candidate fusion event was called with the minimum number of sequenced reads using the default analysis settings (equal to 5 reads). Confirmatory ALK immunohistochemistry or FISH analysis could however not confirm protein overexpression or gene fusion in this case, hence it is considered as a false positive fusion.

Copy number analysis

Copy number data for investigated genes were extracted from 450K Illumina methylation data as described by [6] for 46 Lund tumors. Calls of gain and loss were made using a gain cut-off of log2ratio > 0.1 and a loss cut-off of < –0.1 for segmented data. Amplifications were called as having a segmented log2ratio > 0.5 and verified by visual inspection. Copy number calls for 10 cases from Seidel et al. [5] were extracted from matched Affymetrix SNP6 data as described by [6, 7].

REFERENCES

1. Travis BE W.D., Muller-Hermelink H.K., Harris C.C. World Health Organization Classification of Tumours. Pathology and Genetics of Tumours of the Lung, Pleura, Thymus and Heart. 2004. (Lyon: IARC Press).

2. Travis WD, Brambilla E, Noguchi M, Nicholson AG, Geisinger K, Yatabe Y, Powell CA, Beer D, Riely G, Garg K, Austin JH, Rusch VW, Hirsch FR, et al. International Association for the Study of Lung Cancer/American Thoracic Society/European Respiratory Society: international multidisciplinary classification of lung adenocarcinoma: executive summary. Proc Am Thorac Soc. 2011; 8:381–385.
3. Goldstraw P. International Association for the Study of Lung Cancer (IASLC). Staging manual in thoracic oncology. 2009. (Orange Park: Editorial RxPress).
4. Sobin L GM, Wittekind C. International Union Against Cancer (UICC). TNM classification of malignant tumours. 2009. (Chichester: Wiley-Blackwell).
5. CLCGP . A genomics-based classification of human lung tumors. Sci Transl Med. 2013; 5; 209ra13.
6. Karlsson A, Jonsson M, Lauss M, Brunnstrom H, Jonsson P, Borg A, Jonsson G, Ringner M, Planck M, Staaf J. Genome-wide DNA Methylation Analysis of Lung Carcinoma Reveals One Neuroendocrine and Four Adenocarcinoma Epitypes Associated with Patient Outcome. Clin Cancer Res. 2014; 20:6127–6140.
7. Karlsson A, Ringner M, Lauss M, Botling J, Micke P, Planck M, Staaf J. Genomic and transcriptional alterations in lung adenocarcinoma in relation to smoking history. Clin Cancer Res. 2014; 20:4912–4924.

SUPPLEMENTARY FIGURE AND TABLES

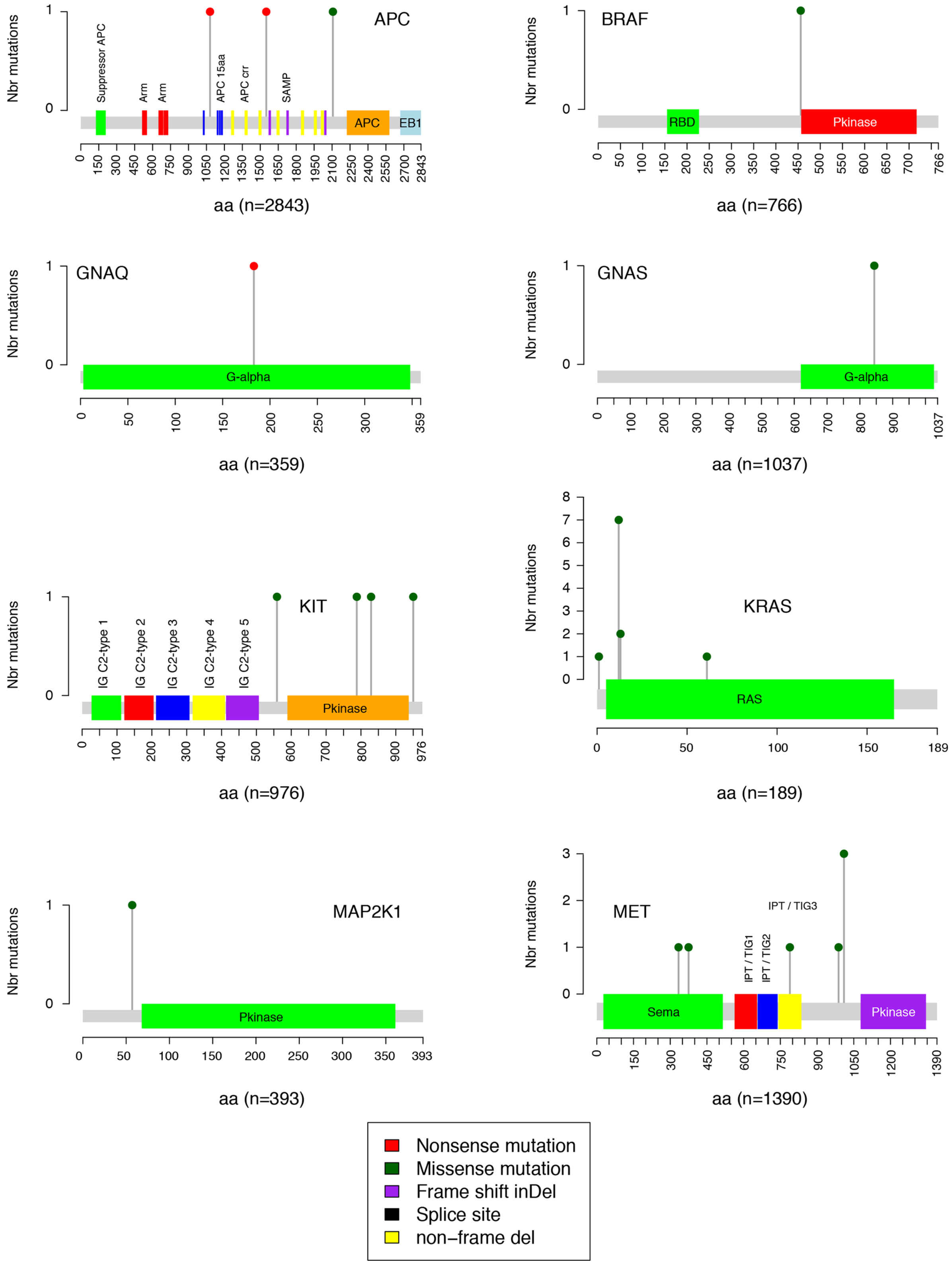

Supplementary Figure S1: Mapping of detected variants in 73 LC and LCNEC cases to functional protein domains for 13 genes. (*Continued*)

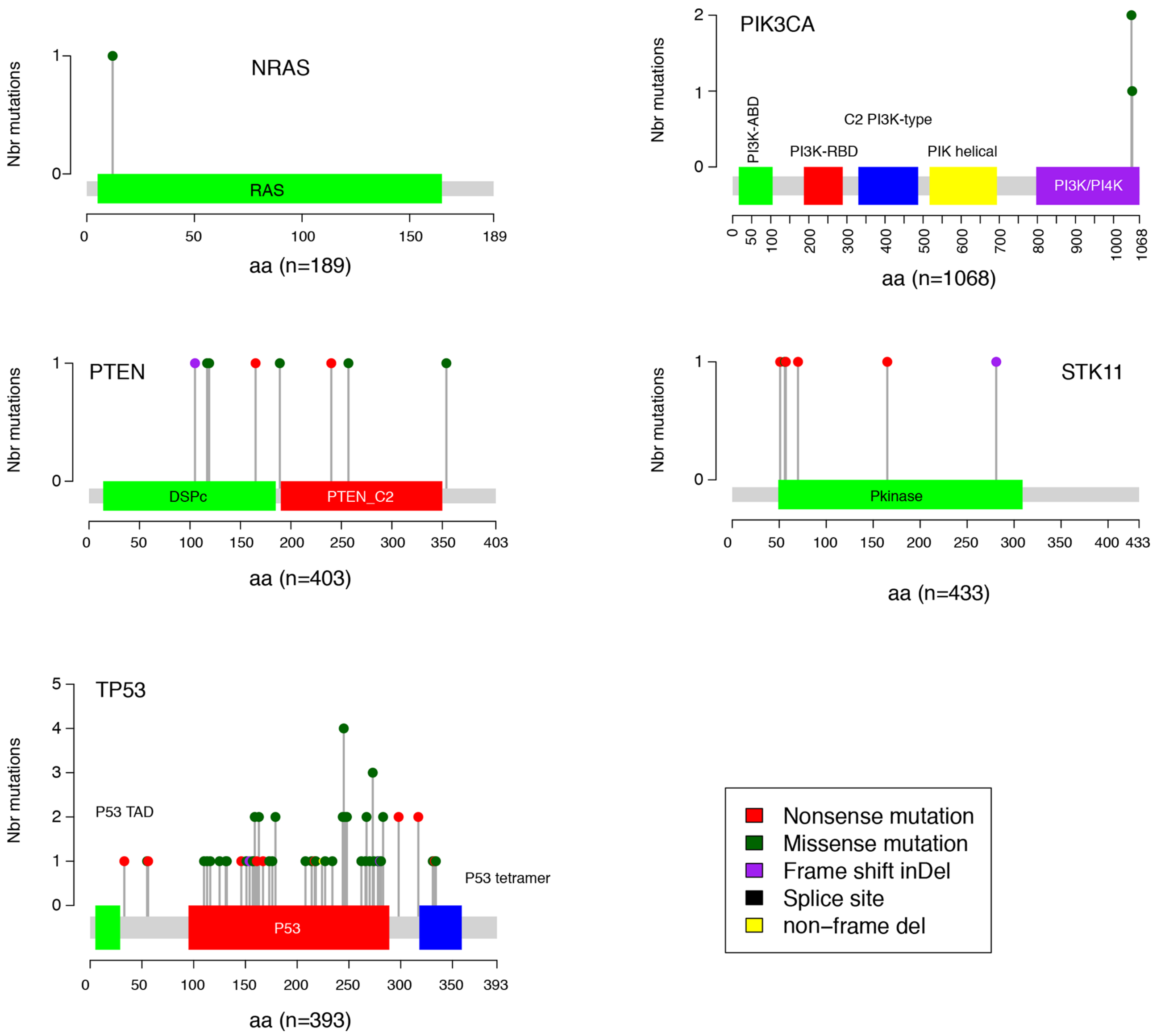

Supplementary Figure S1: (*Continued*) Mapping of detected variants in 73 LC and LCNEC cases to functional protein domains for 13 genes. In each of the 13 lollipop gene plots, the positions of detected variants (excluding splice-site mutations) are plotted against functional protein domains obtained from either Uniprot, or the MutationMapper tool from the CBio Portal. If multiple mutations occur in a gene, all variants are included for the sample. Colored circles indicate the type of mutation and stem height indicate the number of mutations at a given amino acid (aa) position. If more than one mutation type occur at a given amino acid position, the stem and circles represent the cumulative addition of variants.

Supplementary Table S1: Detailed clinicopathological characteristics for Lund cases

Supplementary Table S2: Variants detected in the Lund and Seidel et al. Cohort

Supplementary Table S3: Mutations in subgroups of LC and LCNEC tumors
